# Impacts of Penicillin Binding Protein 2 Inactivation on β-Lactamase Expression and Muropeptide Profile in *Stenotrophomonas maltophilia*

**DOI:** 10.1128/mSystems.00077-17

**Published:** 2017-08-29

**Authors:** Yi-Wei Huang, Yu Wang, Yun Lin, Chin Lin, Yi-Tsung Lin, Cheng-Chih Hsu, Tsuey-Ching Yang

**Affiliations:** a Department of Biotechnology and Laboratory Science in Medicine, National Yang-Ming University, Taipei, Taiwan; b Department of Chemistry, National Taiwan University, Taipei, Taiwan; c School of Public Health, National Defense Medical Center, Taipei, Taiwan; d Division of Infectious Diseases, Department of Medicine, Taipei Veterans General Hospital, Taipei, Taiwan; e School of Medicine, National Yang-Ming University, Taipei, Taiwan; University of California, San Diego

**Keywords:** beta-lactamases, penicillin binding proteins, peptidoglycan

## Abstract

Penicillin binding proteins (PBPs) are involved in peptidoglycan synthesis, and their inactivation is linked to β-lactamase expression in *ampR*–β-lactamase module–harboring Gram-negative bacteria. There are seven annotated PBP genes, namely, *mrcA*, *mrcB*, *pbpC*, *mrdA*, *ftsI*, *dacB*, and *dacC*, in the *Stenotrophomonas maltophilia* genome, and these genes encode PBP1a, PBP1b, PBP1c, PBP2, PBP3, PBP4, and PBP6, respectively. In addition, *S. maltophilia* harbors two β-lactamase genes, L1 and L2, whose expression is induced via β-lactam challenge. The impact of PBP inactivation on L1/L2 expression was assessed in this study. Inactivation of *mrdA* resulted in increased L1/L2 expression in the absence of β-lactam challenge, and the underlying mechanism was further elucidated. The roles of *ampNG*, *ampD*_*I*_ (the homologue of *Escherichia coli ampD*), *nagZ*, *ampR*, and *creBC* in L1/L2 expression mediated by a *ΔmrdA* mutant strain were assessed via mutant construction and β-lactamase activity determinations. Furthermore, the strain *ΔmrdA*-mediated change in the muropeptide profile was assessed using liquid chromatography mass spectrometry (LC-MS). The mutant Δ*mrdA*-mediated L1/L2 expression relied on functional AmpNG, AmpR, and NagZ, was restricted by AmpD_I_, and was less related to the CreBC two-component system. Inactivation of *mrdA* significantly increased the levels of total and periplasmic *N*-acetylglucosaminyl-1,6-anhydro-*N*-acetylmuramyl-l-alanyl-d-glutamyl-*meso*-diamnopimelic acid-d-alanine (GlcNAc-anhMurNAc tetrapeptide, or M4N), supporting that the critical activator ligands for mutant strain Δ*mrdA*-mediated L1/L2 expression are anhMurNAc tetrapeptides.

**IMPORTANCE** Inducible expression of chromosomally encoded β-lactamase(s) is a key mechanism for β-lactam resistance in *Enterobacter cloacae*, *Citrobacter freundii*, *Pseudomonas aeruginosa*, and *Stenotrophomonas maltophilia*. The muropeptides produced during the peptidoglycan recycling pathway act as activator ligands for β-lactamase(s) induction. The muropeptides 1,6-anhydromuramyl pentapeptide and 1,6-anhydromuramyl tripeptide are the known activator ligands for *ampC* β-lactamase expression in *E. cloacae*. Here, we dissected the type of muropepetides for L1/L2 β-lactamase expression in an *mrdA* deletion mutant of *S. maltophilia*. Distinct from the findings with the *ampC* system, 1,6-anhydromuramyl tetrapeptide is the candidate for *ΔmrdA*-mediated β-lactamase expression in *S. maltophilia*. Our work extends the understanding of β-lactamase induction and provides valuable information for combating the occurrence of β-lactam resistance.

## Abstract (precis)

Inducible expression of chromosomally encoded β-lactamase(s) is a key mechanism for β-lactam resistance in *Enterobacter cloacae*, *Citrobacter freundii*, *Pseudomonas aeruginosa*, and *Stenotrophomonas maltophilia*. The muropeptides produced during the peptidoglycan recycling pathway act as activator ligands for β-lactamase(s) induction. The muropeptides 1,6-anhydromuramyl pentapeptide and 1,6-anhydromuramyl tripeptide are the known activator ligands for *ampC* β-lactamase expression in *E. cloacae*. Here, we dissected the type of muropepetides for L1/L2 β-lactamase expression in an *mrdA* deletion mutant of *S. maltophilia*. Distinct from the findings with the *ampC* system, 1,6-anhydromuramyl tetrapeptide is the candidate for *ΔmrdA*-mediated β-lactamase expression in *S. maltophilia*. Our work extends the understanding of β-lactamase induction and provides valuable information for combating the occurrence of β-lactam resistance.

## INTRODUCTION

Peptidoglycan (PG), which is composed of PG monomers, forms a mesh-like layer outside the plasma membrane of bacteria and is a vital component in bacterial survival. The PG monomer consists of disaccharide subunits of *N*-acetylglucosamine and *N*-acetylmuramic acid, with the pentapeptide l-Ala-d-Glu-diaminopimelic acid (DAP)-d-Ala-d-Ala in Gram-negative bacteria. In view of its importance, PG has been recognized as a target for antibiotic development. β-Lactam antibiotics, the most widely used group of antibiotics, inhibit PG biosynthesis via their high affinity for penicillin binding proteins (PBPs) (1).

PBPs are a set of inner membrane-bound enzymes involved in PG synthesis (2). According to their molecular size, PBPs are classified as either high molecular weight (HMW) or low molecular weight (LMW) (3). HMW PBPs are transpeptidases (TPases) and/or transglycosylase (TGases). TGase joins the disaccharide pentapeptides together and forms polysaccharide strands. The adjacent stem pentapeptides from different strands are cross-linked by TPase activity. TPase targets the d-Ala-d-Ala of a donor pentapeptide and forms a peptide bond between the fourth amino acid of the donor peptide and the DAP moiety of the adjacent recipient peptide, with concomitant removal of the terminal d-Ala of the donor pentapeptides. LMW PBPs have dd-carboxylpeptidase (DD-CPase), with the terminal d-Ala removed from pentapeptides. The resulting tetrapeptides are not substrates for TPase, and the cross-linking by TPase is blocked.

Each bacterium harbors an array of PBPs, which catalyze the polymerization of polysaccharide chains and the cross-linking of pentapeptide side chains. The PBP system of *Escherichia coli* is currently the most extensively studied. *E. coli* harbors five HMW PBPs (PBP1a, -1b, -1c, -2, and -3) and eight LMW PBPs (PBP4, -5, -6, -7, and -8 and DacD, AmpC, and AmpH) (4–7). When the PBP activity is inhibited by β-lactam, the PG integrity is compromised, which in turn causes bacterial death.

Bacteria have developed an array of resistance mechanisms to escape attack from β-lactams (8). One of the primary mechanisms leading to β-lactam resistance in some Gram-negative bacteria, for example, *Enterobacter cloacae*, *Citrobacter freundii*, *Pseudomonas aeruginosa*, and *Stenotrophomonas maltophilia*, is the inducible expression of a chromosomally encoded β-lactamase(s) that inactivates β-lactams. When the PBP activity of a bacterium is inhibited by β-lactam, PG turnover is perturbed and certain PG turnover products accumulate in the periplasm. The cell indeed uses this PG signal to induce β-lactamase gene expression as a key resistance mechanism against β-lactam antibiotics (9). Two major mechanisms have been characterized for PBP inactivation-mediated β-lactamase induction: the AmpG-AmpD-NagZ-AmpR circuit in *E. cloacae* (10) and *P. aeruginosa* (11) and the BlrAB/CreBC two-component regulatory system (TCS) in *Aeromonas hydrophilia* (12).

During PG turnover, lytic transglycosylases (LTs) participate in PG cleavage with concomitant formation of a 1,6-anhydro bond at the MurNAc residue of the released muropeptide (13). The degraded sacculi, anhydrodisaccharide peptides, are transported from the periplasm into the cytosol via AmpG permease (14). Once the anhydrodisaccharide peptides are transported into the cytosol, AmpD, a cytoplasmic *N*-acetyl-muramyl-l-alanine amidase, efficiently cleaves the stem peptide from anhydrodisaccharide peptides, and the resulting products are normally recycled into UDP-MurNAc pentapeptide, which acts as a repressor ligand (RL) to inhibit *ampC* expression (15). In the absence of β-lactam, the activity of AmpD is sufficient for hydrolyzing all anhydrodisaccharide peptides produced by normal PG turnover, and then *ampC* expression is repressed. When a bacterium is challenged by β-lactam, the PBP activities are inhibited by β-lactam binding, and surplus anhydrodisaccharide peptides are transported from the periplasm into the cytosol. The AmpD activity becomes saturated, and excess anhydrodisaccharide peptides are processed by NagZ; β-*N*-acetyl-glucosaminidase and the resultant product, 1,6-anhydroMurNAc peptide (anhydromonosaccharide peptides), is the activator ligand (AL) for *ampC* expression (16, 17). The known ALs for *ampC* induction are 1,6-anhydromuramyl pentapeptide and 1,6-anhydromuramyl tripeptide (18, 19).

BlrAB (the homologue of CreBC in *E. coli*), which is involved in β-lactamase expression, has been identified in *A. hydrophilia* (12). The inhibition of the DD-CPase activity of PBP4 by β-lactams results in the accumulation of *N*-acetylglucosaminyl-*N*-acetylmuramyl pentapeptides (disaccharide pentapeptides) in the periplasm, which then induces BlrAB activation and β-lactamase gene expression (12).

*S. maltophilia* is an important nosocomial pathogen and is regarded as a reservoir of antibiotic resistance determinants (20). Intrinsic resistance to β-lactams occurs via the induction of chromosomally encoded L1 and L2 β-lactamases (21). In general, the L1/L2 induction in *S. maltophilia* is similar to that in *E. cloacae* and *P. aeruginosa*, via the *ampG-ampD-nagZ-ampR* regulatory circuit, with a few minor differences. First, instead of AmpG permease, an AmpN-AmpG permease system is responsible for the transport of degraded PG sacculi in *S. maltophilia* (22). Second, *ampD*_*I*_, but not *ampD*_*II*_, has been shown to be relevant to β-lactamase expression, although two *ampD* homologues, *ampD_I_* and *ampD_II_*, have been identified in the *S. maltophilia* genome (23). Third, we have demonstrated that disruption of *mrcA* (encoding PBP1a) results in an increase in basal L1/L2 activity in a NagZ-independent manner (24, 25). Therefore, in addition to the known NagZ-dependent pathway, a NagZ-independent pathway for L1/L2 expression exists in *S. maltophilia*. The involvement of a TCS in PBP inactivation-mediated β-lactamase expression has not been reported so far in *S. maltophilia*.

Each bacterium has its own unique PBP system. Different β-lactams exhibit distinct affinities toward individual PBPs in different microorganisms. Therefore, determination of the relationship between PBP inactivation and β-lactamase induction potential in individual microorganisms is of great importance. Here, we further investigated the roles of other PBPs, in addition to PBP1a, in L1/L2 expression in *S. maltophilia*. We found that PBP2 inactivation confers increased L1/L2 basal activity in the absence of β-lactam challenge. Furthermore, we tried to elucidate the possible AL responsible for strain *ΔmrdA*-mediated L1/L2 expression. A more complete understanding of the circuits regulating β-lactamase expression could lead to the identification of potential targets for controlling β-lactamase-mediated resistance and preservation of the antibacterial activities of the β-lactam class of antibiotics.

## RESULTS

### Analysis of putative PBP genes in *S. maltophilia* K279a.

Genome-wide bioinformatics analysis of the *S. maltophilia* K279a genome (26) in comparison with the known *E. coli* PBPs led to the identification of gene sequences with the closest homologues in the *S. maltophilia* genome. These genes were Smlt3826, Smlt3681, Smlt3602, Smlt4056, Smlt0750, Smlt0462, and Smlt4050, the orthologues for the *mrcA*, *mrcB*, *pbpC*, *mrdA*, *ftsI*, *dacB*, and *dacC E. coli* genes, respectively. *S. maltophilia* lacks clear homologues for *E. coli dacA*, *dacD*, and *pbpG*. The predicted PBPs of *S. maltophilia* shared 36 to 47% identity and 53 to 63% similarity with those of *E. coli* (see Table S1 in the supplemental material).

10.1128/mSystems.00077-17.7 TABLE S1  Seven annotated PBPs of *S. maltophilia* K279a and their homologues in *E. coli*. Download TABLE S1, TIF file, 0.5 MB. Copyright © 2017 Huang et al. 2017 Huang et al. This content is distributed under the terms of the Creative Commons Attribution 4.0 International license.

### Basal and cefuroxime-induced β-lactamase activities of PBP mutants.

To determine involvement of the putative PBP proteins in β-lactamase activity in *S. maltophilia* KJ, each PBP mutant of *S. maltophilia* was constructed, yielding KJΔ*mrcA* (PBP1a mutant) (24), KJΔ*mrcB* (PBP1b mutant), KJΔ*pbpC* (PBP1c mutant), KJΔ*mrdA* (PBP2 mutant), KJΔ*dacB* (PBP4 mutant), and KJΔ*dacC* (PBP6 mutant). After several attempts, we were unable to produce the PBP3 mutant, which might have been the result of the essentiality of the PBP3 protein for the viability of *S. maltophilia*.

**FIG 1:**
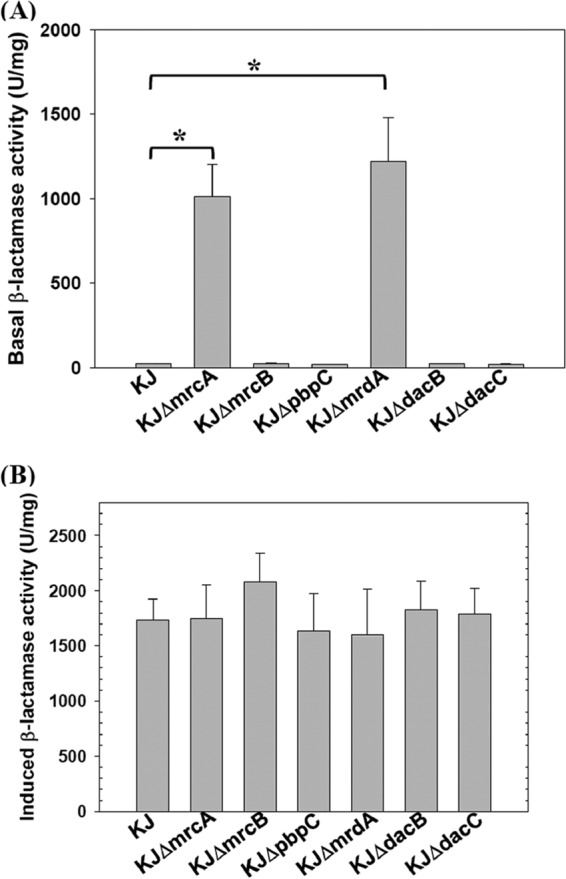
Impact of PBP inactivation on basal and induced β-lactamase activities in *S. maltophilia* strains. The strains assayed were the wild-type KJ and its derived PBP in-frame deletion mutants. Data are the means of three independent experiments. Error bars indicate the standard deviations for three triplicate samples. *, *P* ≤ 0.001 (Student’s *t* test). (A) Basal lactamase activity. Overnight cultures assayed were inoculated into fresh LB with an initial OD_450_ of 0.15. After 3 h of culture, the lactamase activity was determined. (B) Cefuroxime-induced β-lactamase activity. Overnight cultures for the assay were diluted to an optical density at 450 nm of 0.15 and subsequently grown at 37°C for 1 h. Induction was carried out using 30 μg/ml cefuroxime for 2 h.

The impact of each PBP inactivation on the basal and cefuroxime-induced β-lactamase activities was assessed (Fig. 1). *S. maltophilia* KJ harbors two inducible β-lactamases, L1 and L2, and cefuroxime has been proven to be a potent L1/L2 inducer (21). In agreement with previous data (24), the inactivation of *mrcA* caused an increase in basal L1/L2 activity. Likewise, the KJΔ*mrdA* displayed a high basal β-lactamase activity. The basal β-lactamase activities of the KJΔ*mrcB*, KJΔ*pbpC*, KJΔ*dacB*, and KJΔ*dacC* mutant strains were as low as that of wild-type KJ (Fig. 1A). Furthermore, we also compared the β-lactamase activities of the PBP mutants with the wild-type KJ in the presence of cefuroxime. All the PBP mutants tested displayed cefuroxime-induced β-lactamase activities comparable to that of wild-type KJ (Fig. 1B). The subsequent study was focused on elucidating the mechanism of the increased expression of mutant strain *ΔmrdA*-mediated basal β-lactamase.

### Impact of *mrdA* inactivation on bacterial growth and morphology.

**FIG 2:**
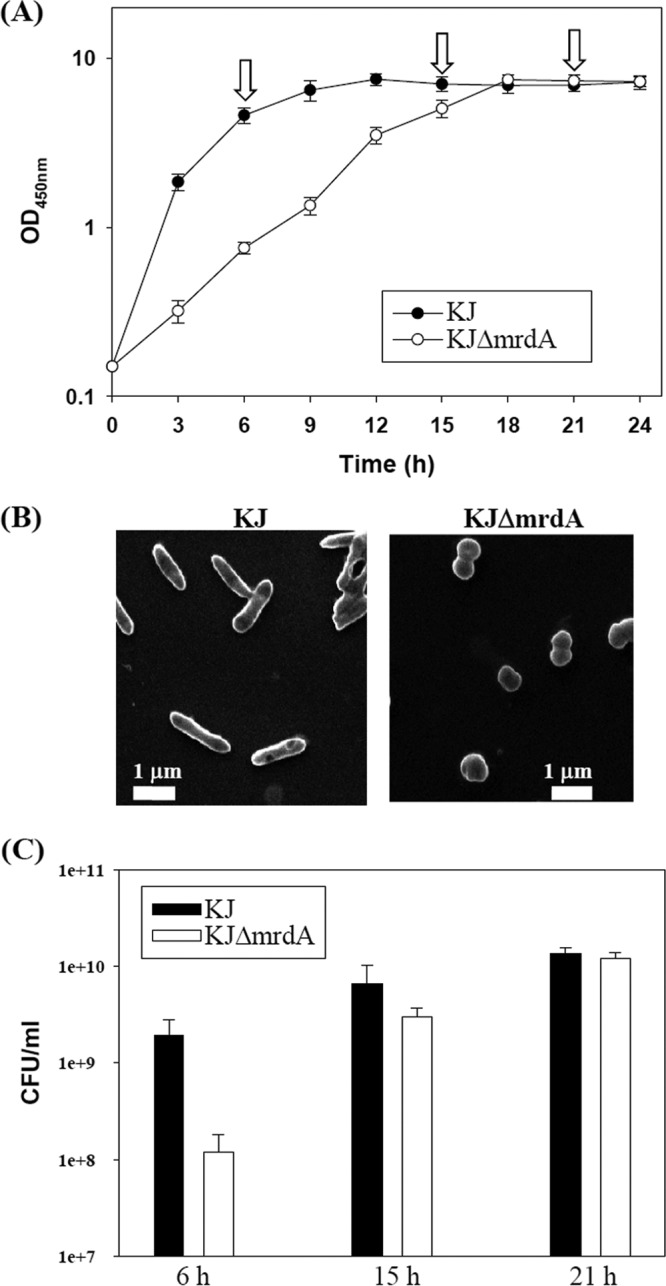
Bacterial growth and morphology of wild-type KJ and the mutant strain KJΔ*mrdA*. (A) Growth curves. Overnight-cultured bacteria were inoculated into fresh LB broth at an initial OD_450_ of 0.15. Bacterial growth was monitored by recording the OD_450_ every 3 h. The 6-h, 15-h, and 21-h bacterial cultures (indicated by arrows) were further assayed for CFU enumeration. Data are the means of three independent experiments. Error bars indicate the standard deviations for three triplicate samples. (B) The morphology of bacteria from exponentially growing cultures was observed by scanning electron microscopy. (C) Bacterial viability from the 6-h, 15-h, and 21-h cultures was monitored by counting the CFU. Data are the means of three independent experiments. Error bars indicate the standard deviations for three triplicate samples.

First, we assessed whether *mrdA* inactivation affected bacterial growth by monitoring the optical density at 450 nm (OD_450_). KJΔ*mrdA* displayed a lower growth rate during exponential growth and an approximately 3-h delay before entering the stationary phase. However, the stationary-phase cells of KJ and KJΔ*mrdA* exhibited equivalent biomass formation (Fig. 2A).

PBP2 is required for rod morphology and cell wall elongation in *E. coli* (27). The KJΔ*mrdA* mutant was therefore evaluated for morphological changes. In contrast to the rod-shaped KJ cells, KJΔ*mrdA* cells were spherical (Fig. 2B). In view of this morphological aberration of strain KJΔ*mrdA*, we wondered whether the difference in the OD_450_ between logarithmic-phase KJ and mutant KJΔ*mrdA* cells was caused by a growth defect or a morphological bias. To address these possibilities, the bacterial growth of strain KJ and KJΔ*mrdA* cells at 6, 15, and 21 h was quantified by CFU enumeration, and the results supported the former possibility. The bacterial growth, evaluated based on either the OD_450_ (Fig. 2A) or CFU enumeration (Fig. 2C), was consistent with the conclusion that the KJΔ*mrdA* deletion mutant has compromised logarithmic-phase growth.

### Impact of *mrdA* inactivation on the expression of L1 and L2.

**FIG 3:**
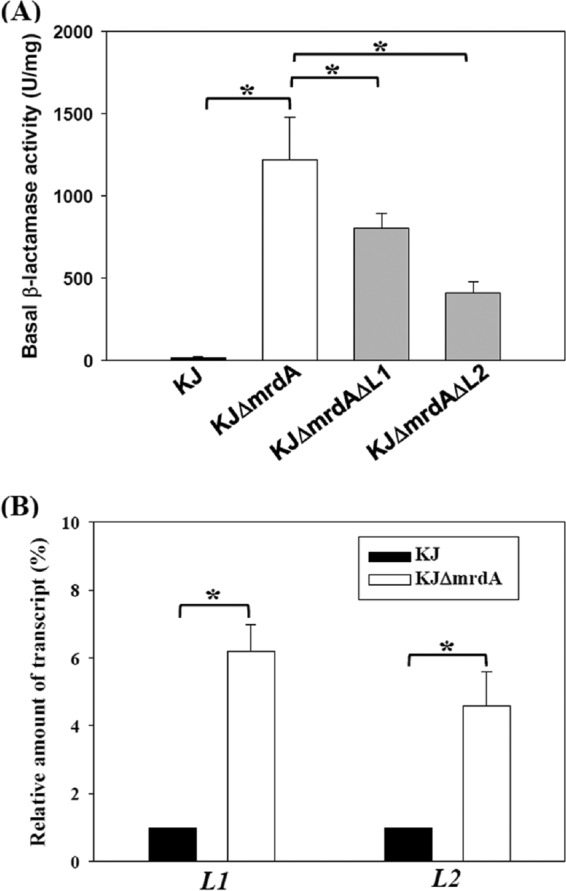
L1 and L2 are attributed to the mutant *ΔmrdA*-mediated β-lactamase activity increase. The strains assayed were the wild-type KJ and its isogenic mutants, KJΔ*mrdA*, KJΔ*mrdA*ΔL1, and KJΔ*mrdA*ΔL2. The overnight cultures assayed were inoculated into fresh LB with an initial OD_450_ of 0.15. After 3 h of culture, the cell pellets were harvested for β-lactamase activity determinations and qRT-PCR. Data are the means of three independent experiments. Error bars indicate the standard deviations for three triplicate samples. *, *P* ≤ 0.001 (Student’s *t* test). (A) The basal lactamase activity. (B) The L1 and L2 transcript levels, determined by qRT-PCR.

There are two active β-lactamases, L1 and L2, in *S. maltophilia* KJ; however, their inducible expression levels appear to be differentially regulated (21). Therefore, we were interested in evaluating the involvement of L1 and L2 in the strain *ΔmrdA*-mediated basal β-lactamase activity increase. The L1 and L2 genes were individually deleted from the KJΔ*mrdA* strain, yielding mutants KJΔ*mrdA*ΔL1 and KJΔ*mrdA*ΔL2. Individual loss of L1 and L2 decreased the basal β-lactamase activity of KJΔ*mrdA* (Fig. 3A). In addition, the L1 and L2 transcripts of KJ and mutant KJΔ*mrdA* cells were also quantified by quantitative reverse transcription-PCR (qRT-PCR). In parallel, the expression levels of L1 and L2 genes in the mutant KJΔ*mrdA* were increased 6.2- ± 0.8-fold and 4.6- ± 1.0-fold (means ± standard deviations) above those in KJ (Fig. 3B), highlighting that strain *ΔmrdA*-mediated increased basal β-lactamase activity is regulated at the transcription level.

### Roles of *ampR*, *ampNG*, *nagZ*, and *ampD*_*I*_ in the mutant *ΔmrdA*-mediated increase in basal β-lactamase activity.

**FIG 4:**
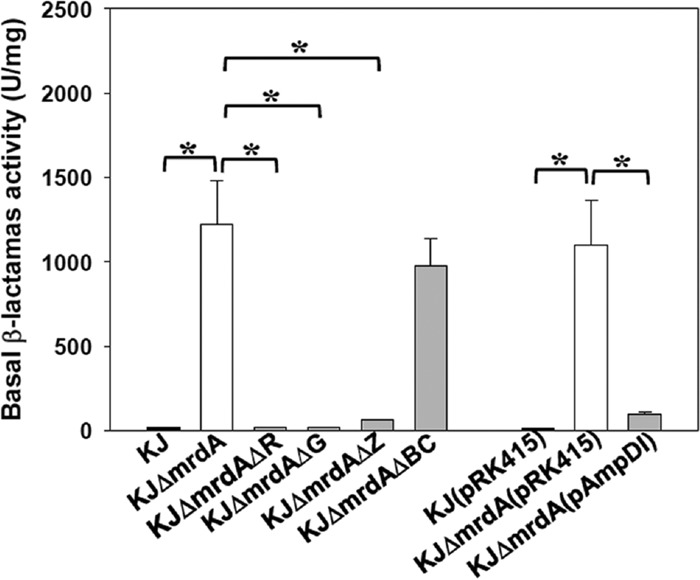
Role of *ampR*, *ampNG*, *nagZ*, *creBC*, and *ampD*_*I*_ in the mutant *ΔmrdA*-mediated β-lactamase expression levels. The overnight-cultured bacteria for the assay were inoculated into fresh LB with an initial OD_450_ of 0.15, cultured for 3 h, and the β-lactamase activities were determined. The data represent means of three repetitions. Error bars indicate the standard deviation for three triplicate samples. *, *P* ≤ 0.01 (Student’s *t* test).

AmpR and AmpNG are essential for β-lactamase expression in *S. maltophilia* (22, 28). We were interested in assessing the essentiality of AmpR and AmpNG in mutant *ΔmrdA*-mediated basal L1/L2 activity. The Δ*ampR* and Δ*ampG* alleles were introduced into strain KJΔ*mrdA*, yielding the double mutants KJΔ*mrdA*Δ*R* and KJΔ*mrdA*Δ*G*, respectively, and these mutants were evaluated for basal L1/L2 activity. Not surprisingly, deletion of *ampR* or *ampG* of strain KJΔ*mrdA* resulted in basal β-lactamase activity at levels comparable to that of the wild-type KJ (Fig. 4), consistent with the known roles of AmpR and AmpNG.

To assess the impact of *nagZ* on the mutant *ΔmrdA*-mediated basal L1/L2 activity, the *nagZ* allele was deleted from the chromosome of strain KJΔ*mrdA*, yielding the mutant KJΔ*mrdA*Δ*Z*. The basal β-lactamase activity of KJΔ*mrd*AΔ*Z* reverted to nearly the same level as that of wild-type KJ (Fig. 4), indicating that strain Δ*mrdA*-mediated basal L1/L2 activity is NagZ dependent.

The CreBC TCS has been shown to be responsible for strain *ΔdacB*-mediated β-lactam resistance increases in *P. aeruginosa* (29), srtrain *ΔdacB*-mediated β-lactamase increases in *A. hydrophilia* (12), and strain Δ*mltD1*-mediated L1/L2 expression increases in *S. maltophilia* (30). To elucidate whether the mutant *ΔmrdA*-mediated basal L1/L2 activity increase is related to the *creBC* TCS, the *ΔcreBC* allele was introduced into strain KJΔ*mrdA*, yielding the mutant KJΔ*mrdA*Δ*BC*, and its basal β-lactamase activity was determined. Figure 4 demonstrates that the majority of strain *ΔmrdA*-mediated basal L1/L2 activity is CreBC independent.

AmpD_I_ overexpression is known to attenuate the β-lactam-induced and mutant strain *ΔmrcA*-mediated basal β-lactamase activity in *S. maltophilia* (23, 24). An *ampD*_*I*_-containing plasmid (pAmpDI) was introduced into strain KJΔ*mrdA* to assess the role of AmpD_I_ in *ΔmrdA*-mediated basal β-lactamase activity. The basal β-lactamase activity of mutant strain KJΔ*mrdA* reverted to the level of wild-type KJ when AmpD_I_ was overexpressed (Fig. 4), supporting that the stem peptide is critical for the activity of the AL generated by *mrdA* inactivation.

### Impact of *mrdA* inactivation on muropeptide profiles.

On the basis of the known AmpC paradigm, the exact AL of ΑmpC expression involves muropeptides, and these AL precursors are transported from the periplasm to the cytosol by AmpG permease (14). Because the roles of AmpG, NagZ, AmpR, AmpD_I_, and CreBC in strain *ΔmrdA*-mediated L1/L2 expression are very similar to those of their corresponding homologues in the AmpC induction model, we hypothesized that the AL for L1/L2 expression are muropeptides, and their precursors should accumulate at high levels in the *ΔmrdA* mutant. To evaluate this, we set to determine the muropeptide profile of PG in wild-type KJ and KJΔ*mrdA.*

**FIG 5:**
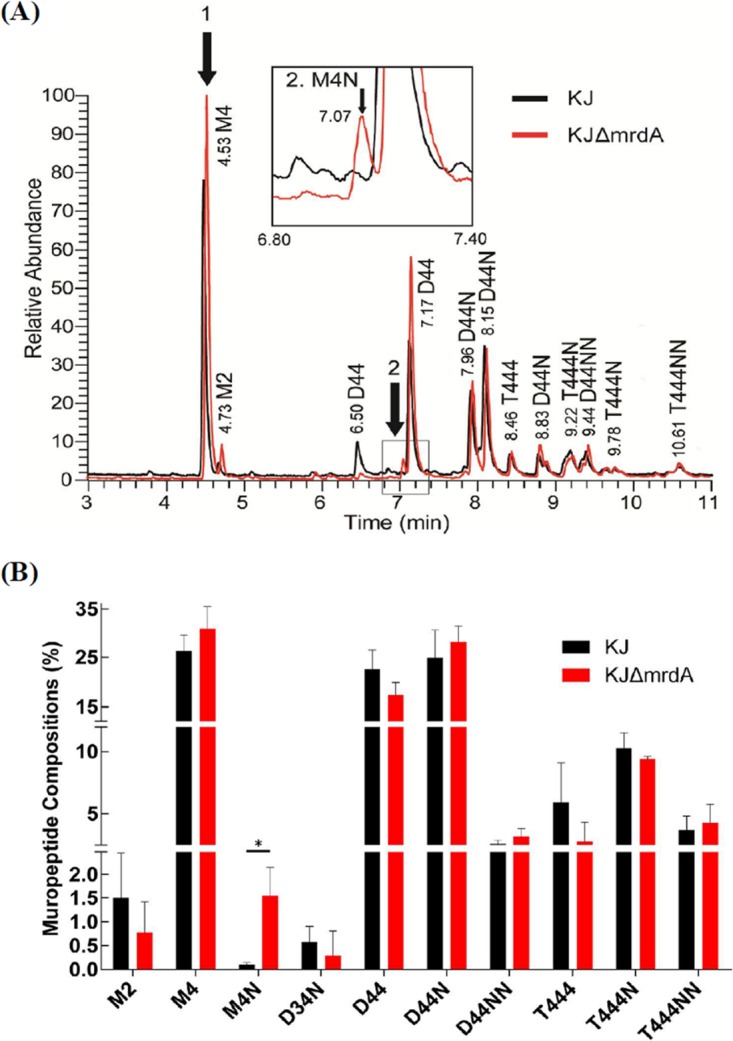
LC-MS analysis results for total muropeptides from wild-type KJ and the *mrdA* mutant, KJΔ*mrdA*. (A) The LC-MS total ion chromatogram (TIC) of wild-type KJ and mutant KJΔ*mrdA*, determined via reversed-phase ultraperformance liquid chromatography coupled to a high-resolution hybrid Orbitrap mass spectrometer. (Inset) Enlargement of the region where M4N is eluted. (The extracted ion chromatogram [EIC] of M4 and M4N are shown in Fig. S3 in the supplemental material.) (B) The ratios of the top 10 muropeptide compositions in wild-type KJ and KJΔ*mrdA* mutant strain. (The full list of identified muropeptides is provided in Table S2). *, *P* < 0.05 (for M4N). Muropeptide symbols: M, monomer; D, dimer; T, trimer (numbers following the letters are the number of amino acid stem peptides). (G) Additional glycines at the end of stem peptides (replacing l-alanine). N, terminating anhydro-muropeptide; dAc, no acetyl group in GluNAc site. The muropeptides that have never been reported in the literature were further validated by MS/MS analysis (Fig. S4 to S6).

Two different protocols were used for muropeptide preparation to clarify the muropeptide content in different cellular locations, as described in Materials and Methods. First, muropeptides were isolated from total cell extracts via muramidase treatment, representing the total muropeptides in murein, periplasm, and cytosol. The total muropeptides and their relative abundance levels were analyzed using liquid chromatography and mass spectrometry (LC-MS) (31–34). Compared to the wild-type KJ, strain KJΔ*mrdA* showed a clear increase in two peaks in the total muropeptide ion chromatogram, labeled as peak 1 (retention time of 4.53) and peak 2 (retention time of 7.07) in Fig. 5A. The corresponding compounds for peaks 1 and 2 were identified as the reduced form of *N*-acetylglucosaminyl-*N*-acetylmuramyl-l-alanyl-d-glutamyl-*meso*-diaminopimelic acid-d-alanine (GlcNAc-MurNAc-tetrapeptide) (M4) and *N*-acetylglucosaminyl-1,6-anhydro-*N*-acetylmuramyl-l-alanyl-d-glutamyl-*meso*-diaminopimelic acid-d-alanine (GlcNAc-anhMurNAc-tetrapeptide) (M4N), respectively (see Fig. S1 and S2 for tandem mass analysis results). The ratio of muropeptide composition is summarized in Fig. 5B and Table S2. The most notable increase in strain KJΔ*mrdA* was in M4N, which increased by ∼10-fold, whereas M4 muropeptide showed a less significant increase in strain KJΔ*mrdA*.

10.1128/mSystems.00077-17.1 FIG S1  Tandem MS analysis results for rM4. (A) The MS2 spectrum of rM4 (parent ion *m/z* 942.42). (B) The MS3 spectrum of the *m/z* 739.34 fragment observed in panel A and the fragmentation map. Download FIG S1, TIF file, 1.7 MB. Copyright © 2017 Huang et al. 2017 Huang et al. This content is distributed under the terms of the Creative Commons Attribution 4.0 International license.

10.1128/mSystems.00077-17.2 FIG S2  Tandem MS analysis results for M4N. (A) The MS2 spectrum of M4N (parent ion *m/z* 922.39). (B) The MS3 spectrum of the *m/z* 719.31 fragment observed in panel A and the fragmentation map. Download FIG S2, TIF file, 1.7 MB. Copyright © 2017 Huang et al. 2017 Huang et al. This content is distributed under the terms of the Creative Commons Attribution 4.0 International license.

10.1128/mSystems.00077-17.8 TABLE S2  Total muropeptides of *S. maltophilia* KJ and the mutant KJΔ*mrdA* analyzed by LC-MS. Download TABLE S2, TIF file, 1.3 MB. Copyright © 2017 Huang et al. 2017 Huang et al. This content is distributed under the terms of the Creative Commons Attribution 4.0 International license.

10.1128/mSystems.00077-17.3 FIG S3  LC-MS analysis results for total muropeptides from wild-type KJ and the *mrdA* mutant, KJΔ*mrdA*. The extracted ion chromatogram (EIC) of wild-type KJ (black) and the *mrdA* mutant, KJΔmrdA (red) had *m/z* values of 471.67 to 471.75 rM4, (rM4 + 2H)^2+^ ions; *m/z* 719.27 to 719.35 (M4N), corresponding to (M4N + H – GlcNAc)^+^ ions. As rD44 (the oxidative form of D44) easily renders the *m/z* 719 fragment, the minor peak in EIC of wild-type KJ at ∼6.45 min is denoted as the in-source fragmentation of D44*. Download FIG S3, TIF file, 1.1 MB. Copyright © 2017 Huang et al. 2017 Huang et al. This content is distributed under the terms of the Creative Commons Attribution 4.0 International license.

10.1128/mSystems.00077-17.4 FIG S4  Tandem MS analysis results for rD04. (A) The MS2 spectrum of rD04 (parent ion *m/z* 710.80) at a retention time of 5.94 with NCE of 10%. (B) The MS2 spectrum of rD04 (parent ion *m/z* 710.80) at a retention time 6.17 with NCE of 10%. Download FIG S4, TIF file, 1.7 MB. Copyright © 2017 Huang et al. 2017 Huang et al. This content is distributed under the terms of the Creative Commons Attribution 4.0 International license.

10.1128/mSystems.00077-17.5 FIG S5  Tandem MS analysis results for rD24. (A) The MS2 spectrum of rD24 (parent ion *m/z* 810.84) at a retention time 6.88. (B) The MS2 spectrum of rD24 (parent ion *m/z* 810.84) at a retention time of 7.35. Download FIG S5, TIF file, 1.7 MB. Copyright © 2017 Huang et al. 2017 Huang et al. This content is distributed under the terms of the Creative Commons Attribution 4.0 International license.

10.1128/mSystems.00077-17.6 FIG S6  Tandem MS analysis results for rT444NN. The typical MS2 spectrum of rT444NN (parent ion *m/z* 917.39) with an NCE of 10%. Download FIG S6, TIF file, 1 MB. Copyright © 2017 Huang et al. 2017 Huang et al. This content is distributed under the terms of the Creative Commons Attribution 4.0 International license.

**FIG 6:**
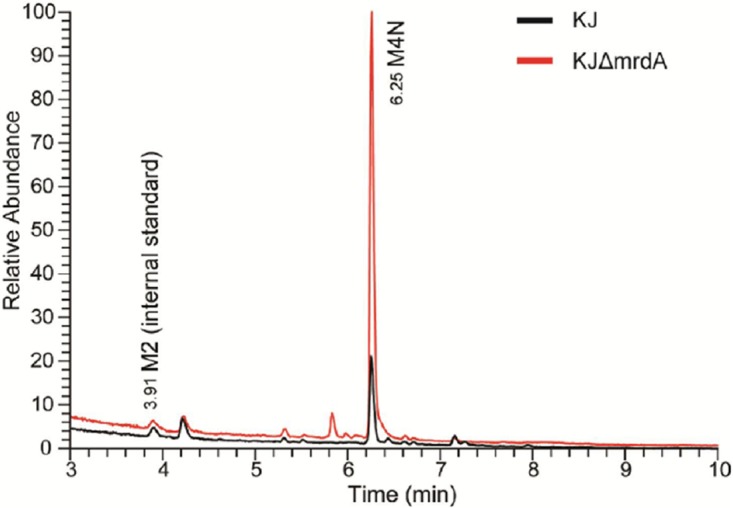
Quantitative LC-MS analysis of periplasmic muropeptides from wild-type KJ and *mrdA* mutant strain KJΔ*mrdA*. The LC-MS total ion chromatograms (TICs) of periplasmic muropeptide analysis of wild-type KJ and the *mrdA* mutant strain, KJΔ*mrdA* were determined by reverse-phase ultraperformance liquid chromatography coupled to a high-resolution hybrid Orbitrap mass spectrometer. A full list of periplasmic muropeptides is provided in Table S3. An equal amount of purified *N*-acetylglucosaminyl-*N*-acetylmuramyl-l-alanyl-d-glutamic acid in its reduced form (GlcNAc-MurNAc dipeptide; M2), which was only observed in total muropeptides (Fig. 5), was spiked in both wild-type KJ and the KJΔ*mrdA* strain as an internal standard to quantify the periplasmic muropeptides.

Based on inferences from the known AmpC and L1/L2 induction models (9, 20), we assumed that M4N is the major AL precursor, transported from the periplasm into the cytosol by the AmpN/AmpG permease system, and further processed into the anhMurNAc tetrapeptides by NagZ in an *ΔmrdA* mutant. To support this assumption, we isolated the periplasmic muropeptides without muramidase treatment. Compared to that of wild-type KJ, the periplasmic muropeptides profile of KJΔ*mrdA* had a considerable increase (∼5-fold) in M4N (Fig. 6), consistent with the findings for the total muropeptides profiles of strains KJ and KJΔ*mrdA*.

10.1128/mSystems.00077-17.9 TABLE S3  Periplasmic muropeptides of *S. maltophilia* KJ and the mutant strain KJΔ*mrdA* analyzed by LC-MS. Download TABLE S3, TIF file, 2.2 MB. Copyright © 2017 Huang et al. 2017 Huang et al. This content is distributed under the terms of the Creative Commons Attribution 4.0 International license.

## DISCUSSION

PBPs, the target of β-lactams, are enzymes responsible for PG synthesis. HMW-PBPs are known to be the primary targets of β-lactam (35), but LMW-PBPs are thought to participate in sensitizing bacteria to β-lactams (36). Therefore, a β-lactam can simultaneously inhibit HMW- and LMW-PBP, resulting in a synergistic bactericidal effect. However, for *ampR*–β-lactamase–harboring Gram-negative bacteria, the loss of PBP activity by β-lactam binding may produce surplus or different muropeptide intermediates involved in chromosomal β-lactamase expression. β-Lactams, which can simultaneously bind to the HMW-PBPs and LMW-PBPs, display strong β-lactamase induction potency and cause high β-lactam resistance; for example, imipenem or cefoxitin is a potent inducer of β-lactamase in *E. cloacae* and *P. aeruginosa* (10). Thus, an effective β-lactam for infection treatment can also be a potent inducer of β-lactam resistance. Because the extent of inactivation of each PBP by different β-lactams varies, β-lactam treatment-mediated β-lactamase expression is complex, and the extent of inactivation of each PBP and the individual impact of PBP inactivation on induced β-lactamase activity are difficult to evaluate. Thus, PBP in-frame deletion mutants obtained via genetic manipulation in the laboratory can be used as an alternative strategy, and this method has been extensively applied in numerous studies (24, 29). Two reports have linked single PBP gene inactivation to increased basal β-lactamase activity in *ampR*–β-lactamase–harboring Gram-negative bacteria, including *dacB* inactivation in *P. aeruginosa* (mutant PAΔ*dacB*) (29) and *mrcA* inactivation in *S. maltophilia* (mutant KJΔ*mrcA*) (24). In this study, we used a novel example, KJΔ*mrdA* (with an *mrdA* in-frame deletion mutant of *S. maltophilia*).

*Escherichia coli* AmpG (AmpG_EC_) shows a strict substrate restriction. The principal requirement for AmpG_EC_ uptake is the presence of anhydrodisaccharide, GlcNAc-anhMurNAc, but the presence of peptides is not necessary. Thus, anhydrodisaccharide and anhydrodisaccharide peptides are compatible substrates for AmpG_EC_ [for example, GlcNAc-anhMurNAc (M0N), GlcNAc-anhMurNAc tripeptide (M3N), GlcNAc-anhMurNAc tetrapeptide (M4N), and GlcNAc-anhMurNAc pentapeptide (M5N)] (14). In addition, the ALs for *E. cloacae* AmpC expression are anhMurNAc tripeptide and anhMurNAc pentapeptide (18, 19). Therefore, for AmpC expression in *E. cloacae*, the precursors of ALs are transported by AmpG from the periplasm to the cytosol and further processed by NagZ in the cytosol. Sufficient cytosolic AmpD activity hydrolyzes AL precursors, prevents formation of ALs, and abolishes AmpC expression. A similar AmpC expression model was also proposed for *P. aeruginosa*, as the components involved in AmpC expression in *P. aeruginosa* are functionally the same as those in *E. coli* (29), although the exact structures of AL precursors and ALs remain unknown in the *P. aeruginosa* system. The information regarding L1/L2 expression is limited compared to the that for the AmpC model, but the following findings provide some clarification for L1/L2 expression in *S. maltophilia*. (i) Distinct from the AmpG in the AmpC model, the permease system for AL precursor transportation is the AmpN-AmpG system in *S. maltophilia* (22). In our previous study, we showed that an intact *ampN-ampG* operon is essential for L1/L2 expression; furthermore, AmpG_EC_ can complement the L1/L2 expression by the *ampN-ampG* mutant in *S. maltophilia* (22). Therefore, it is reasonable to speculate that the AL precursors for L1/L2 expression are generated in the periplasm, with the structural feature of anhydrodisaccharide with tripeptides, tetrapeptides, or pentapeptides, and then transported by AmpNG permease. (ii) There are two reported PBP inactivation mutants associated with L1/L2 upregulation in the *S. maltophilia* model: one is the *mrcA* mutant (24), and the the other is the *mrdA* mutant in or study. A critical distinction between the two mutants is the contribution of NagZ to L1/L2 upregulation. Basal L1/L2 expression in the *ΔmrcA* mutant is NagZ independent (25), but such expression in the *ΔmrdA* mutant is NagZ dependent. We suggest that there are at least two different ALs, AL1 and AL2, for L1/L2 expression in *S. maltophilia*. AL1 is NagZ dependent, while AL2 is not (25). In our study, we further linked AL1 to anhMurNAc tetrapeptides in a mutat *ΔmrdA* background, and the AL1 precursor was found to be GlcNAc-anhMurNAc tetrapeptides (M4N), which accumulate in th periplasm of strain KJΔ*mrdA* cells. Here, we still cannot immediately rule out the possibility that anhMurNAc tripeptide and anhMurNAc pentapeptide are the functional NagZ-dependent ALs for L1/L2 expression of *S. maltophilia*, as in the AmpC model for *E. cloacae* (9, 19). Nevertheless, we are convinced that anhMurNAc tetrapeptides, rather than anhMurNAc tripeptide and anhMurNAc pentapeptide, are the key ALs for muan *ΔmrdA*-mediated L1/L2 expression.

PBP2 possesses TPase, which cross-links the adjacent stem pentapeptides from different strands and maintains the bacterial rod shape. Consistent with the deletion study findings in *E. coli* (37) and *P. aeruginosa* (38), the *mrdA* deletion mutant of *S. maltophilia* displayed a round morphology (Fig. 2B), supporting that PBP2 functions mainly in cross-linking of PG during the bacterial elongation stage. The substrate for PBP2 is the d-Ala-d-Ala moiety of pentapeptides of a PG monomer. In contrast, the terminal d-Ala of pentapeptides is also a target for LMW-PBP action, as LWM-PBP are a type of DD-CPase, which removes the terminal d-Ala from pentapeptides in PG. Because the terminal d-Ala is a substrate for both TPase and DD-CPase, PBP2 inactivation may spare more substrates for DD-CPase. This may explain why the total and periplasmic M4N muropeptides in *mrdA* mutants were much more abundant than those in wild-type KJ (Fig. 5).

**FIG 7:**
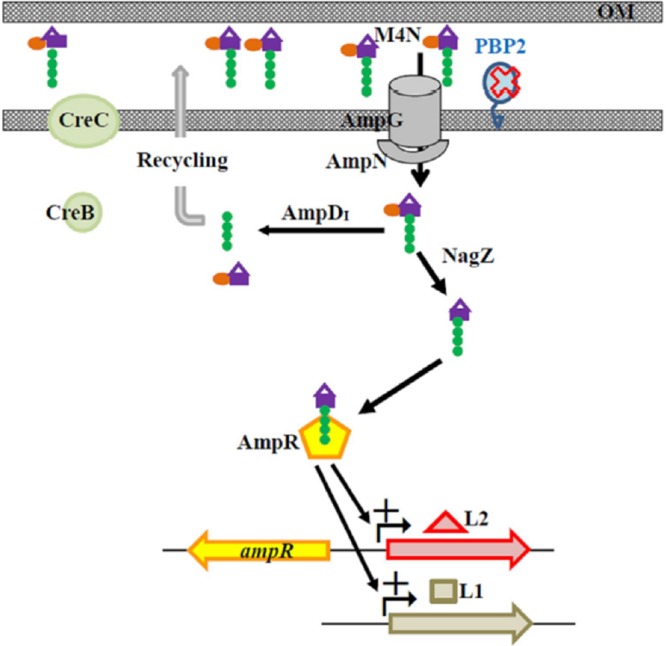
Proposed model of PBP2 inactivation-mediated L1 and L2 expression in *S. maltophilia*. Inactivation of *mrdA* results in the accumulation of GlcNAc-anhMurNAc tetrapeptide (M4N) in the periplasm. M4N is transported from the periplasm into the cytosol by the AmpN/AmpG permease system. The imported M4N is hydrolyzed into GlcNAc-anhMurNAC and free tetrapeptide by AmpD_I_, and the resulting products are further recycled into UDP-MurNAc pentapeptide, which is introduced into the PG monomer biosynthetic pathway. Once the AmpD_I_ activity is saturated, surplus M4N is processed by NagZ, generating the AL, anhMurNAc tetrapeptide. The AL binds to the transcriptional regulator AmpR and converts AmpR into an activator for L1 and L2 expression.

The results of this study led to the development of a model of muropeptide turnover and L1/L2 expression in a mutant *ΔmrdA* background (Fig. 7). Inactivation of PBP2 increases the accumulation of M4N in the periplasm. M4N is transported into the cytosol via the AmpN/AmpG permease system. AmpD_I_ hydrolyzes the imported M4N into GlcNAc-anhMurNAC and free tetrapeptide, which are typically recycled into UDP-MurNAc pentapeptide for use in PG monomer biosynthesis. When AmpD_I_ activity is saturated by M4N, the surplus M4N is further processed by NagZ to form the exact AL, anhMurNAc tetrapeptides, to induce L1/L2 expression in the presence of a functional AmpR. When excess AmpD_I_ activity is exogenously introduced, M4Ns are completely hydrolyzed by AmpD_I_ and no ALs are formed, explaining why no β-lactamase activity is detectable in strain KJΔ*mrdA*(pAmpDI). Furthermore, the CreBC TCS of *S. maltophilia* shows a low response to the muropeptide of M4N. Collectively, strain *ΔmrdA*-mediated L1/L2 expression depends on functional AmpG, AmpR, and NagZ, is constrained by AmpD_I_ activity, and is less related to the CreBC TCS. This is supported by our β-lactamase activity determinations (Fig. 4) and muropeptide profiles (Fig. 5 and 6).

## MATERIALS AND METHODS

### Construction of in-frame deletion mutants.

*S. maltophilia* in-frame deletion mutants were created using a double-crossover homologous recombination system as previously described (23). The recombinant plasmids pΔmrcB, pΔpbpC, pΔmrdA, pΔdacB, and pΔdacC were prepared for mutant constructions. Two DNA fragments targeting the 5′-terminus and 3′-terminus of the mutated PBP gene were obtained by PCR using the primer sets MrcBN-F/MrcBN-R and MrcBC-F/MrcBC-R for pΔmrcB, PbpCN-F/PbpCN-R and PbpCC-F/PbpCC-R for pΔpbpC, MrdAN-F/MrdAN-R and MrdAC-F/MrdAC-R for pΔmrdA, DacBN-F/DacBN-R and DacBC-F/DacBC-R for pΔdacB, and DacCN-F/DacCN-R and DacCC-F/DacCC-R for pΔdacC (Table S4). These PCR amplicons were digested and subsequently cloned into pEX18Tc (39). The recombinant plasmid mobilization, transconjugant selection, and mutant confirmation steps were performed as described previously (23). The double mutant was constructed from the single mutant sequentially through the same procedure.

10.1128/mSystems.00077-17.10 TABLE S4  PCR primers used in this study. Download TABLE S4, TIF file, 1.9 MB. Copyright © 2017 Huang et al. 2017 Huang et al. This content is distributed under the terms of the Creative Commons Attribution 4.0 International license.

### Determination of β-lactamase activity.

β-Lactamase induction was studied using a published procedure (25) with nitrocefin (100 μM) as the substrate. Enzyme activity was calculated by using a molar absorption coefficient for nitrocefin of 20,500 M^−1^ cm^−1^ at 486 nm. The specific activity was expressed as the nanomoles of nitrocefin hydrolyzed per minute per milligram of protein. The protein content was determined with the Bio-Rad protein assay reagent, with bovine serum albumin as a standard. All experiments were performed in triplicate.

### Bacterial growth.

Overnight cultures were inoculated into fresh LB broth with an initial OD_450_ of 0.15. Cell growth was monitored by recording the OD_450_ at an interval of 3 h. Cells from the time points of 6, 15, and 21 h were serially diluted and plated in duplicate on LB agar. After overnight incubation at 37°C, the number of colonies formed was counted and recorded.

### Scanning electron microscopy.

The preparation of bacterial specimens was carried out as previously described (40). Then, bacterial morphology was examined by using a high-resolution FEI Inspect S scanning electron microscope.

### Preparation of muropeptides. (i) Total muropeptides.

Total muropeptides were isolated as described previously with some modifications (41). A 100-ml mid-logarithmic-phase bacterial culture was harvested by centrifugation, resuspended in 3 ml of phosphate-buffered saline buffer, and dropped into a boiling 6-ml 6% SDS solution. After boiling for 15 h, the murein saccule pellets were collected by ultracentrifugation (40,000 × *g* for 20 min at room temperature), washed free of SDS with Milli-Q water, and digested with pronase (100 μg/ml in 10 mM Tris-HCl [pH 7.2], 0.06% [wt/vol] NaCl) for 4 h at 60°C. The reaction was inactivated by boiling the sample for 30 min. Samples were pelleted by ultracentrifugation (40,000 × *g* for 20 min at room temperature), resuspended in 200 μl of 50 mM sodium phosphate buffer (pH 4.9), and digested with muramidase (40 μg/ml) for 16 h at 37°C. Muramidase digestion was stopped by incubation at 100°C for 10 min. The coagulated proteins were removed by centrifugation at 14,000 rpm for 10 min. The muropeptides in supernatant were reduced by using 100 mM sodium borate and 20 mg/ml sodium borohydride in 0.5 M borate buffer (pH 9.0) for 3 h. Finally, the sample was adjusted to pH 3 to 4 by using 50% (vol/vol) orthophosphoric acid.

### (ii) Periplasmic muropeptides.

Periplasmic muropeptides were prepared as described previously with some modifications (42). Cells from 30-ml mid-logarithmic-phase bacterial cultures were harvested by centrifugation. The cell pellets were washed twice with phosphate-buffered saline buffer, suspended in 1 ml of hypertonic solution (40% sucrose in 10 mM sodium phosphate buffer [pH 7.4]), and then incubated on ice for 20 min. After centrifugation at 12,000 rpm for 10 min, cell pellets were resuspended into 200 μl of hypotonic solution (10 mM sodium phosphate buffer [pH 7.4]) and incubated on ice for an additional 20 min. Cells were pelleted at 12,000 rpm for 10 min, and the supernatant was labeled as the periplasmic fraction. The periplasmic muropeptides were reduced by 100 mM sodium borate and 20 mg/ml sodium borohydride in 0.5 M borate buffer (pH 9.0) for 3 h. Finally, the sample was adjusted to pH 3 to 4 with 50% (vol/vol) orthophosphoric acid.

### (iii) M2 muropeptides.

The total muropeptides samples were pooled and subjected to LC separation/purification to collect individual muropeptide species. The Waters 600 HPLC system consists of a 996 photodiode array detector, 600 solvent pump, and 600 controller and was used to separate the muropeptides. The mobile phases A and B were ultrapure water and acetonitrile, respectively, and both were added with 0.1% (vol/vol) trifluoroacetic acid. The LC elution condition was slightly different based on the pump pressure of the HPLC system. After balancing the Unitary C18 column (100 Å, 5 µm, 10 mm by 250 mm) with 5.0% mobile phase B for 30 min, a 2.0-ml sample was injected into the sample loop. The elution started with 5.0% mobile phase B for 15.0 min, which was then raised to 14.0% mobile phase B in 30.0 min, and the system was washed with 95.0% mobile phase B for 10.0 min. The fractions were collected every 30 s. The purity of each fraction was checked with a Q Exactive Plus hybrid quadrupole-orbitrap mass spectrometer (Thermo Fisher Scientific, USA). M2 was dissolved in ultrapure water and the stock was held at −80°C until use for standards for the quantitative analysis of periplasmic muropeptides.

### LC-MS analysis of muropeptides. (i) Total muropeptides.

The total muropeptides sample was centrifuged at 14,000 rpm for 30 min, and the supernatant was diluted 10-fold with H_2_O for subsequent LC-MS analysis.

A Dionex UltiMate 3000 UHPLC system coupled with a Q Exactive Plus hybrid quadrupole-orbitrap mass spectrometer (Thermo Fisher Scientific, USA) was used for LC-MS analysis. The mobile phases A and B were ultrapure water and acetonitrile, respectively, and both were added with 0.1% (vol/vol) formic acid. After balancing the Acquity UPLC CSH C18 column (130 Å, 1.7 µm, 2.1 mm by 100 mm) with mobile phase A, 3 μl of sample was injected into the sample loop. The percentage of the mobile phase B was increased to 3.0% within 1 min, then raised to 12.0% in 7 min. Within 4 min, mobile phase B was raised to 13.5% and then raised rapidly to 90.0% mobile phase B within 1 min and left for 3 min. The column temperature was controlled at 52°C in the whole analysis program. The capillary temperature of Q Exactive Plus was set at 360°C. The Thermo HESI-II probe was used as the ion source, and the spray voltage was set at 3.5 kV, with the auxiliary gas temperature set at 406°C. For MS1 acquisition, the resolution was set with 35,000, and the mass range was from 450 to 2,000 *m/z* at positive mode. Data-dependent tandem MS (MS/MS) was then performed for further structural validation of the peptidoglycans. A full MS spectrum (450 to 2,000 *m/z*, 35,000 resolution) with a 100-ms maximum injection time followed by MS1-dependent MS/MS acquisition which consisted of 10 scans in a cycle with a Δ*m*/*z* 3 isolation window (17,500 resolution) with 120-ms maximum injection time. The top 10 most abundant peaks in the MS1 scan were sequentially isolated and fragmented by higher-energy collision dissociation (HCD) using nitrogen as the collision gas with a normalized collision energy of ∼17%. All the MS spectra were recorded in profile mode.

### (ii) Periplasmic muropeptides.

The periplasmic muropeptide samples were centrifuged at 14,000 rpm for 30 min, and 20-μl volumes of supernatants were mixed with 2 μl of purified M2 as the internal standard for subsequent LC-MS analysis.

The instruments used to analyze periplasmic muropeptides sample were the same as the total muropeptides sample, whereas the elution condition for LC was different. The same CSH C18 column first balanced with 2.0% mobile phase B, and 11-μl sample volumes were injected into the sample loop. The percentage of the mobile phase B was increased to 5.0% from 0.5 to 1.0 min and kept at 5.0% for 1.0 min, then raised to 8.0% in 2.0 min. It was increased to 9.5% B in the next 5.0 min, up to 11.0% B within 1.5 min, and then raised rapidly to 90.0% B within 1.5 min and left for 3 min. The capillary temperature of Q Exactive Plus was set at 253°C. Other instrument settings were the same as those for the LC-MS analysis of total muropeptides.

### Data processing.

A reference table of muropeptides was established for peak assignments in the following manner: (i) a core *m/z* list of all possible monomer, dimer, and trimer muropeptides containing different numbers of stem peptides was first generated and was based on the main structures of monomers of *S. maltophilia* cell wall. (ii) The list was further expanded by including possible modifications of muropeptides, e.g., reduction, stem peptide cross-linking, glycosidic bond cleavage between *N*-acetylglucosamine and *N*-acetylmuramic acid, during the sample preparation. (iii) A list of *m/z* values for possible in-source fragmentation products was added. The theoretical *m/z* and isotopic patterns of all the proposed muropeptides in the reference table were determined with the *ecipex* package and were used to annotate the ion signals obtained by LC-MS (43).

An automatic muropeptide analysis platform based on the R programing language was developed and used to determine the relative levels of each muropeptide species. In short, MSconvert, a free software published by ProteoWizard, was implemented to convert the .raw file to an .mzXML file so as to deconvolute the Thermo RAW file in R. We utilized the *mzR* package to read the raw mass spectra for peak picking in each scan (44), where ions of intensities lower than 1.0 × 10^5^ obtained by Q Exactive Plus mass spectrometers in our experiments were neglected. Ions of the same *m/z* (tolerance, 0.02 *m*/*z*), similar isotope intensity (tolerance, 20%), and same charge state that continuously appeared in more than two successive scans were considered identical. Ions of different retention times were distinguished as different molecular species. Ions were annotated based on their *m/z* and isotopic patterns, as suggested in the reference muropeptides table. Intensities of identical muropeptides of different charge states and their products of in-source fragmentation that appeared at the same retention times were summed up. The areas under the curves (AUCs) of each muropeptide species in the ion chromatograms were calculated and exported as composition ratios. Those AUC values for reduced muropeptides were combined with AUCs for their corresponding parent muropeptides. For total muropeptide analysis, the ratios of the AUCs of each muropeptide and the total AUC of all annotated muropeptides were calculated to determine the relative compositions of each annotated species, whereas for periplasmic muropeptides analysis the ratio of the AUC of each muropeptide to the AUC of rM2 was calculated to determine the quantity of each annotated species. All the LC-MS data reported in the manuscript were manually extracted and inspected meticulously to validate the efficacy of the automatic platform.
