# Supplementary figures and images for: Impacts of Penicillin Binding Protein 2 Inactivation on β-Lactamase Expression and Muropeptide Profile in Stenotrophomonas maltophilia
Source: mSystems. 2017 Aug 29;2(4):e00077-17. doi: 10.1128/mSystems.00077-17 (PMC5574705; doi:10.1128/mSystems.00077-17)

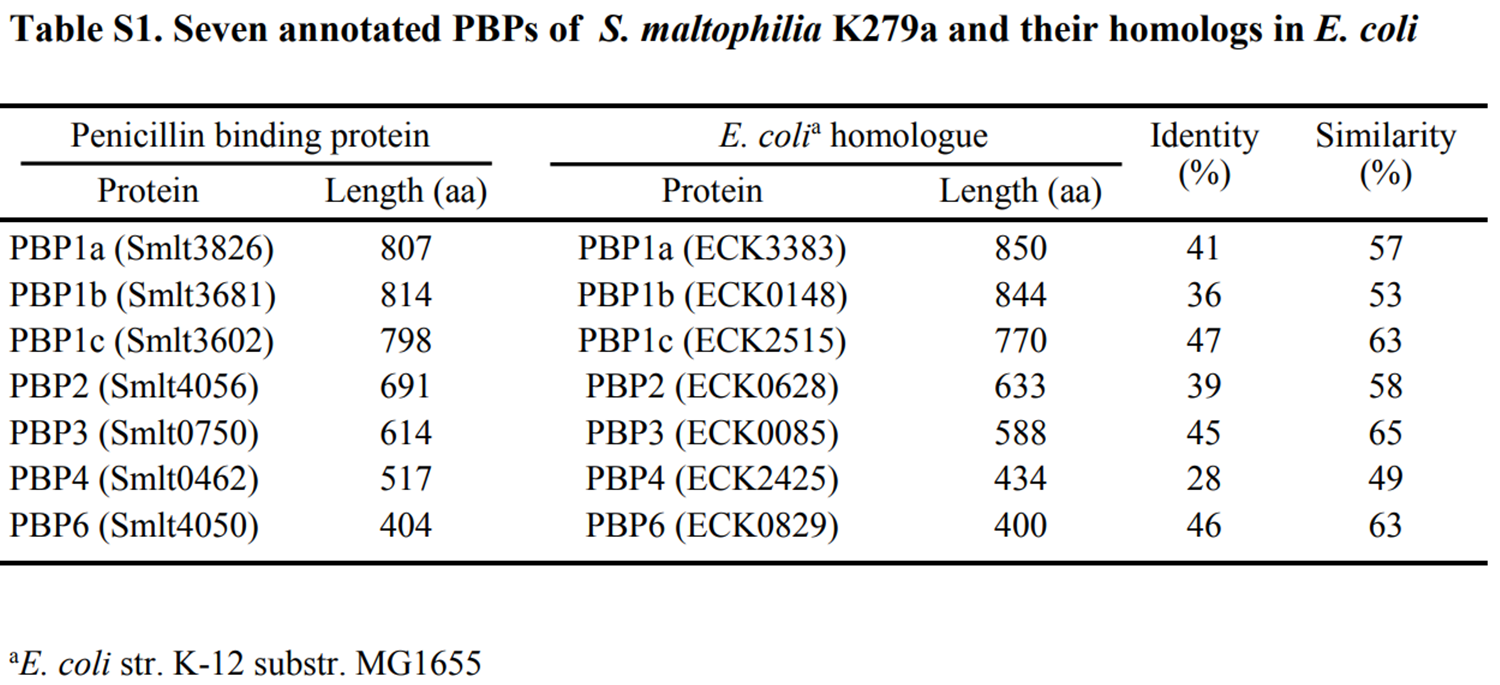

Supplement: TABLE S1 [file sys004172128st1.tif]

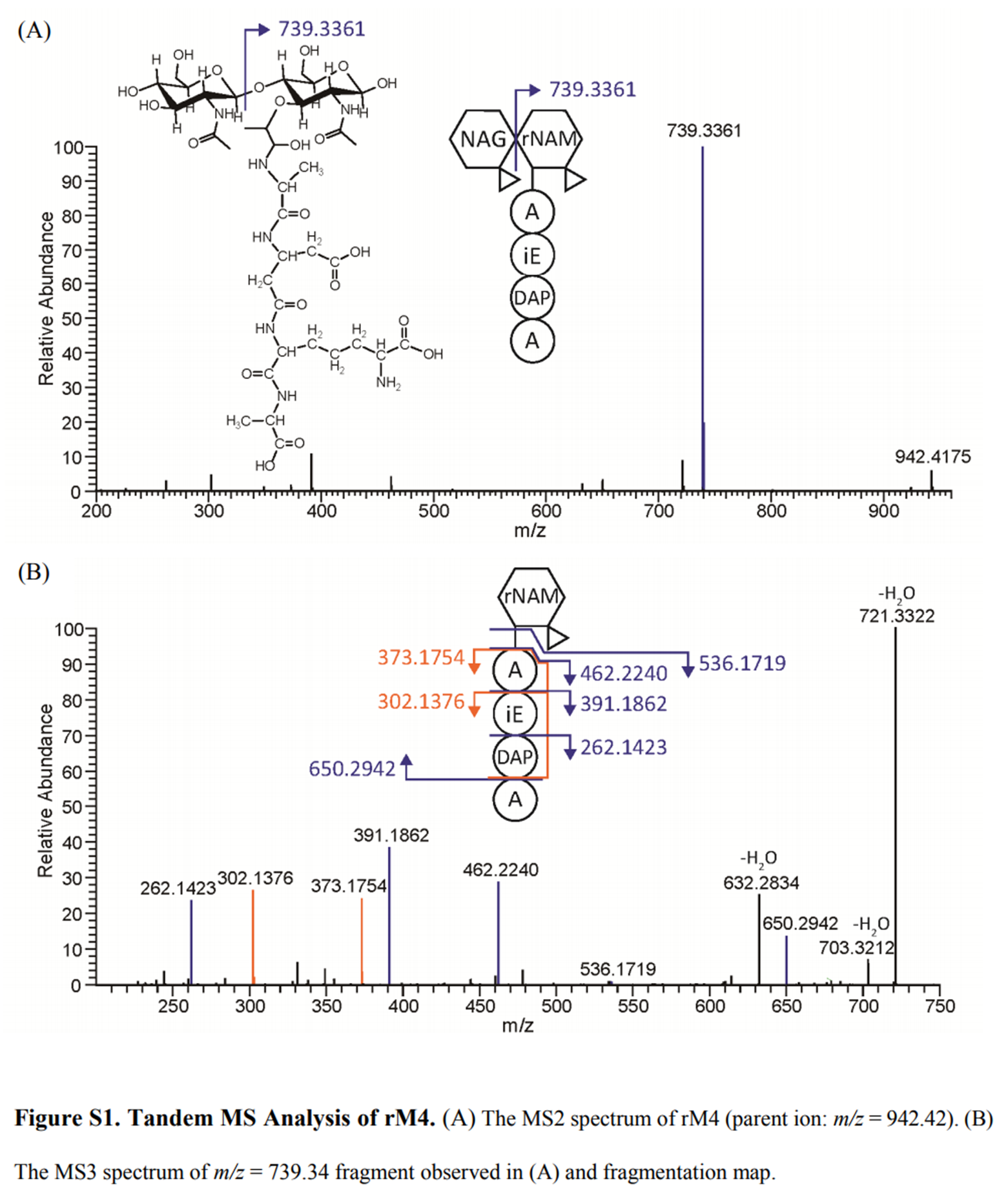

Supplement: FIG S1 [file sys004172128sf5.tif]

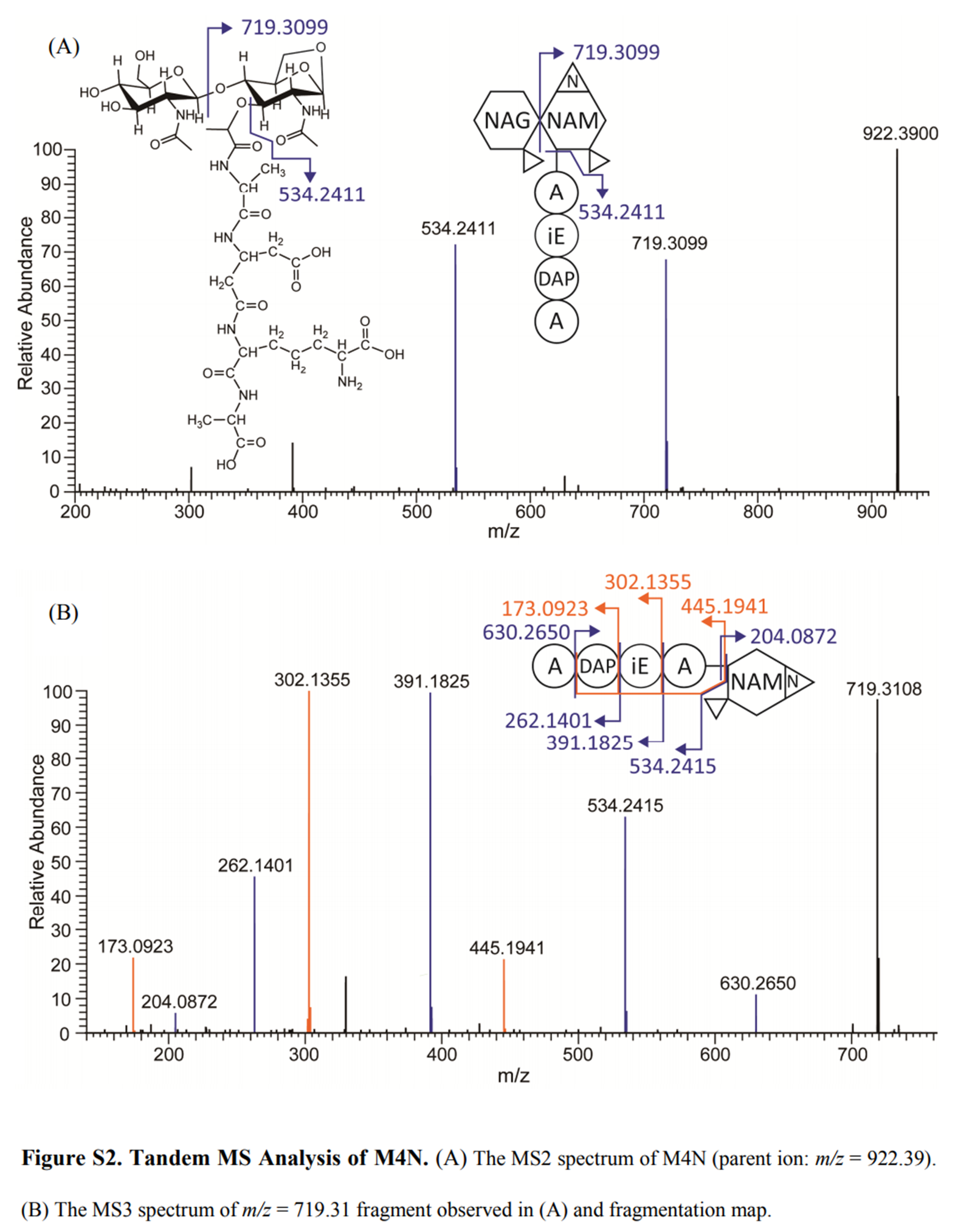

Supplement: FIG S2 [file sys004172128sf6.tif]

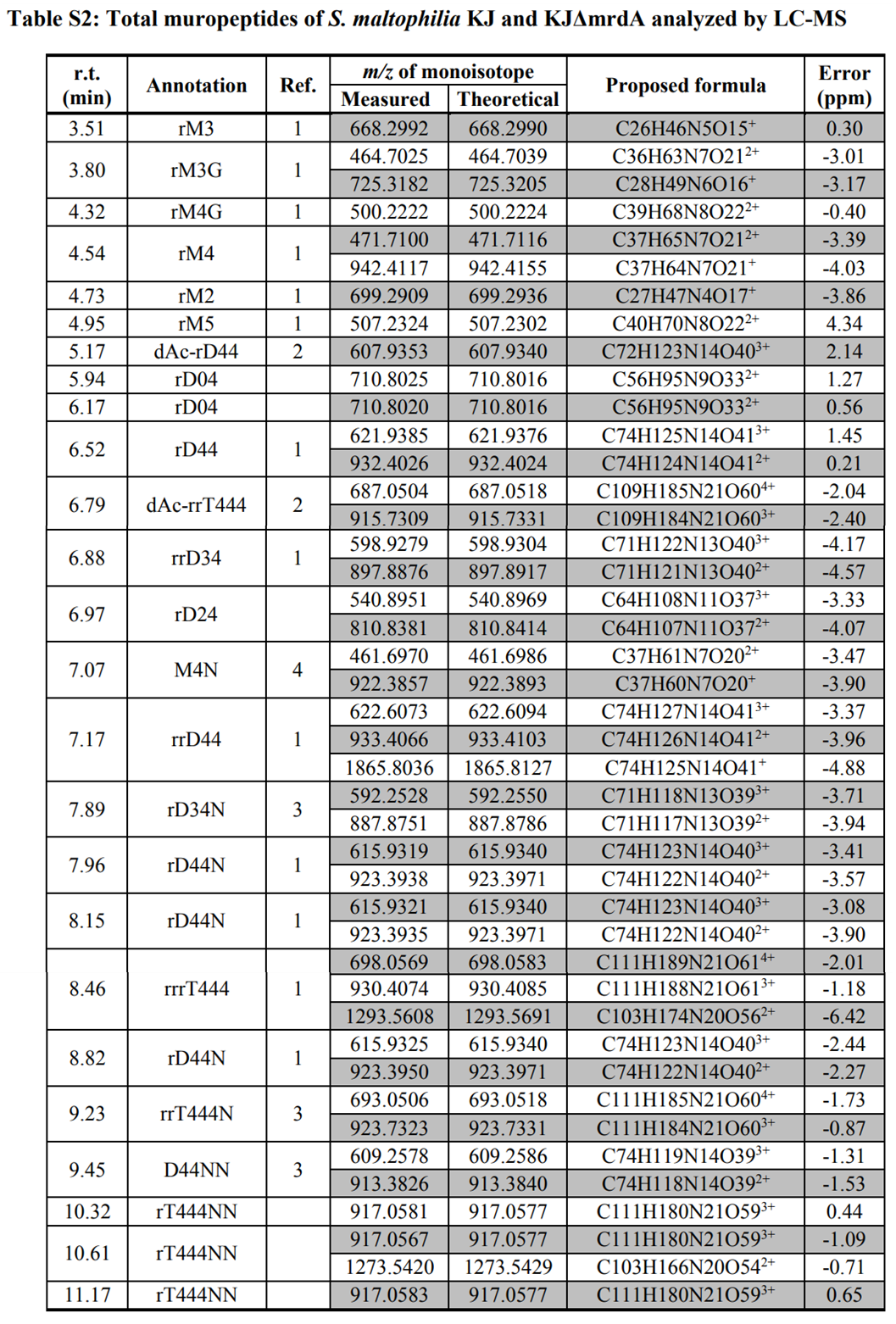

Supplement: TABLE S2 [file sys004172128st2.tif]

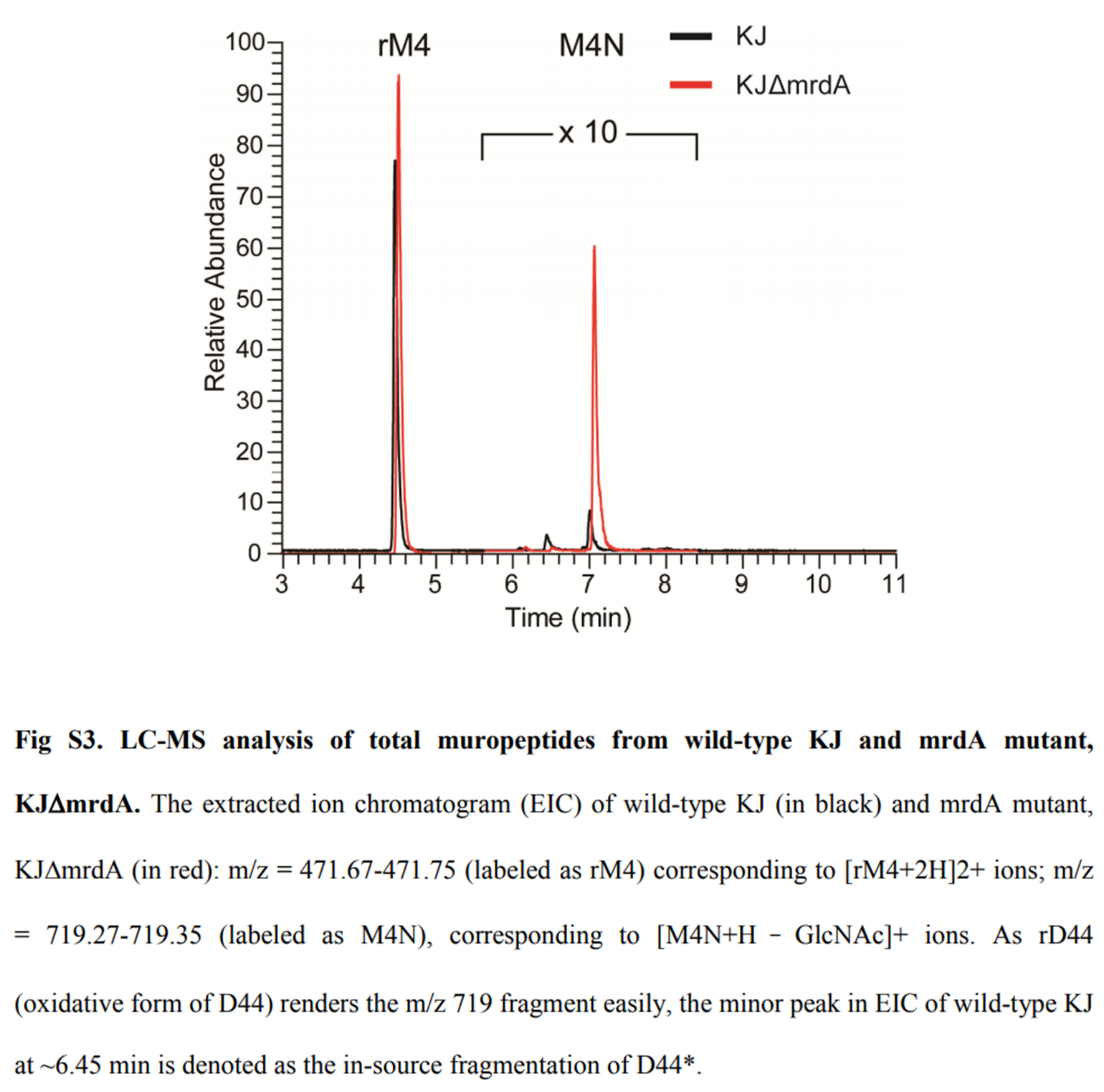

Supplement: FIG S3 [file sys004172128sf7.tif]

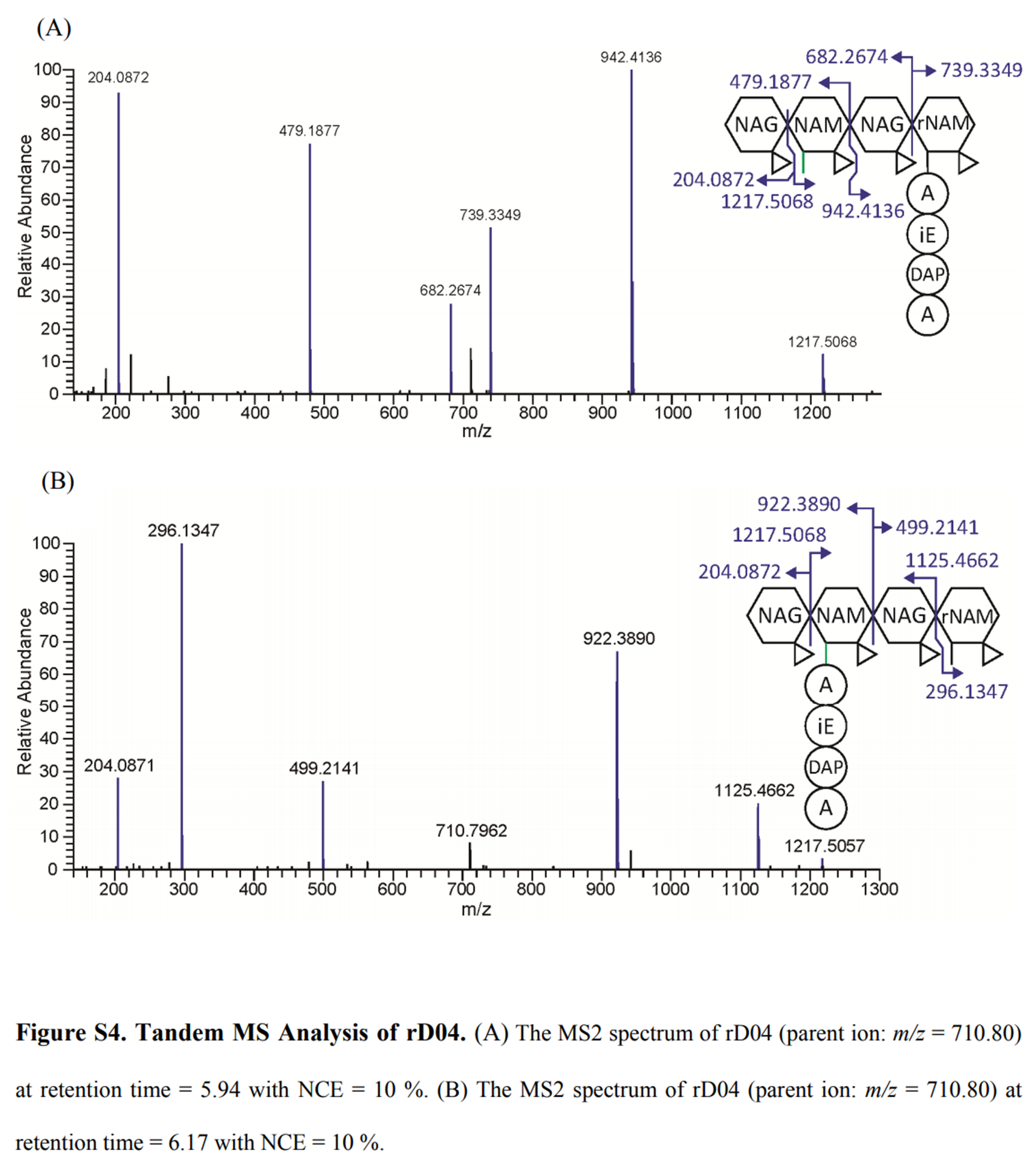

Supplement: FIG S4 [file sys004172128sf8.tif]

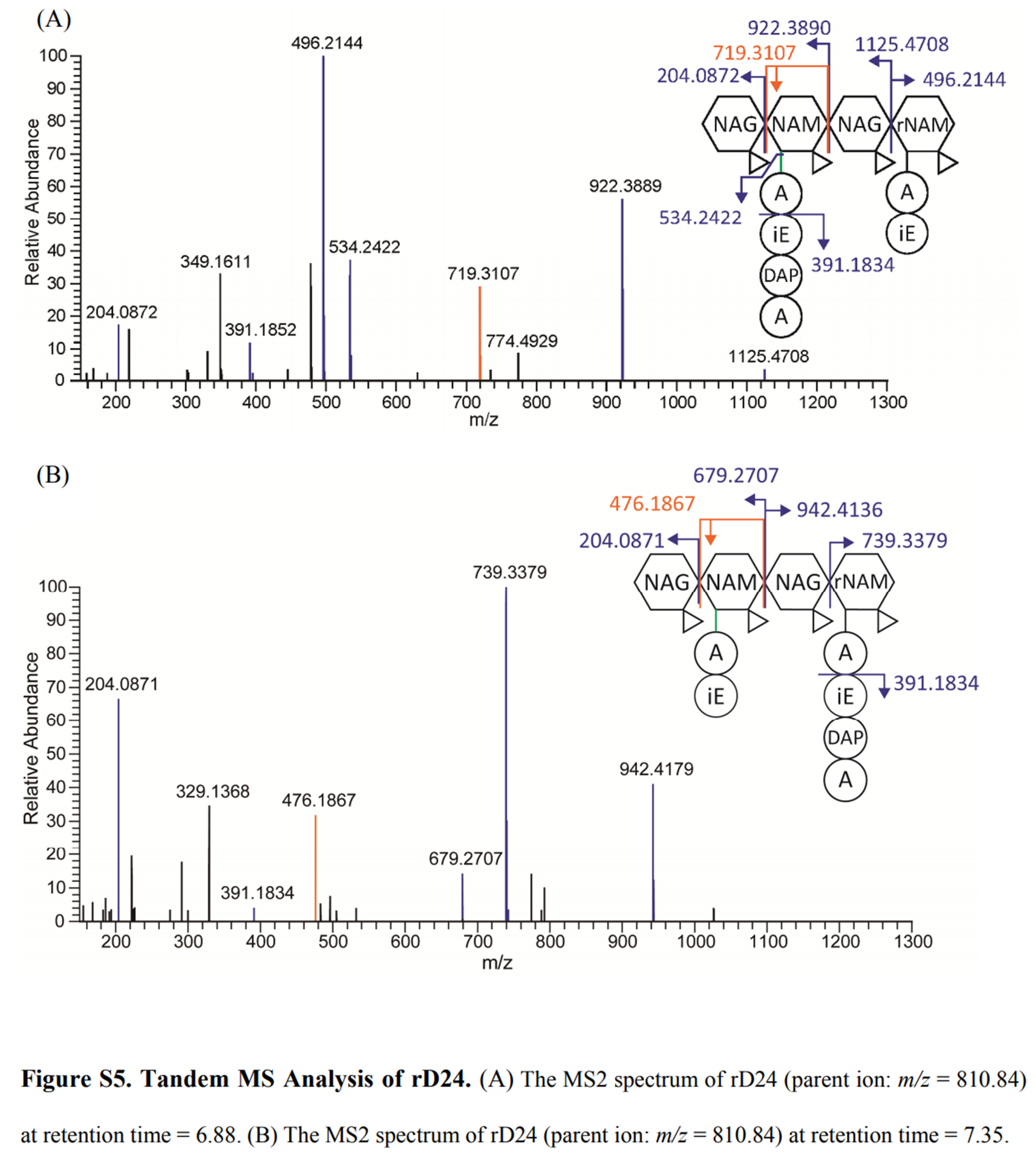

Supplement: FIG S5 [file sys004172128sf9.tif]

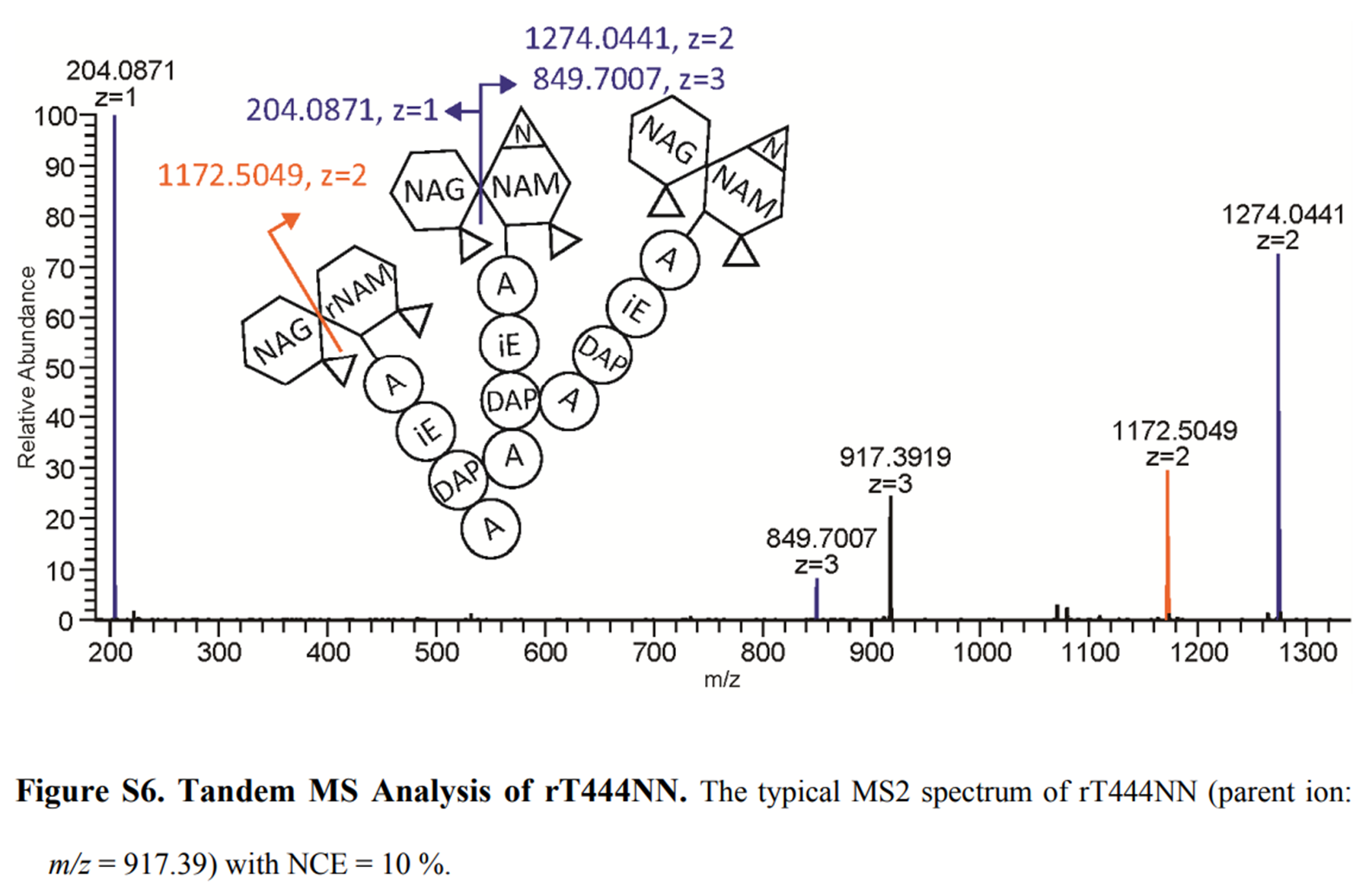

Supplement: FIG S6 [file sys004172128sf10.tif]

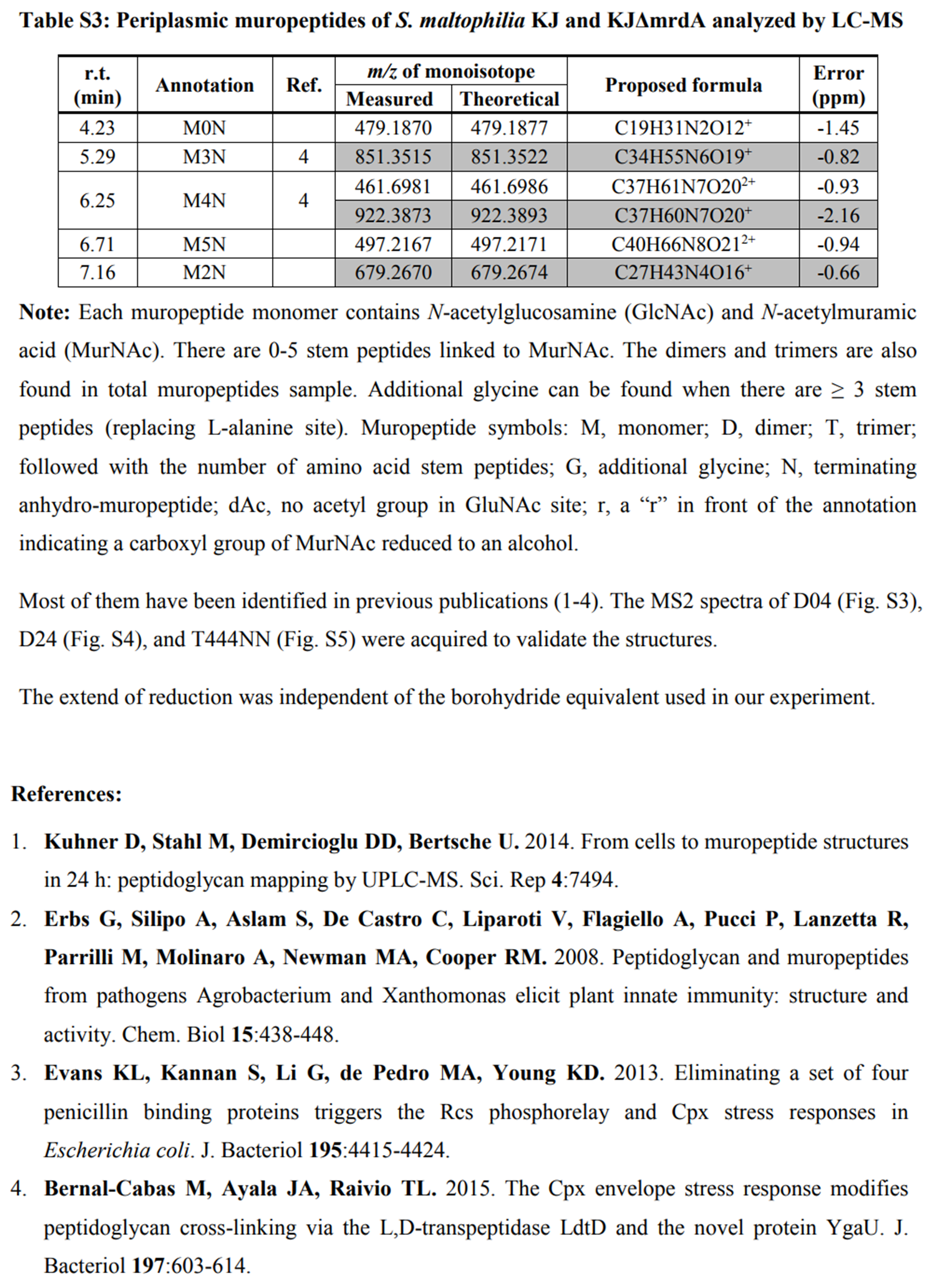

Supplement: TABLE S3 [file sys004172128st3.tif]

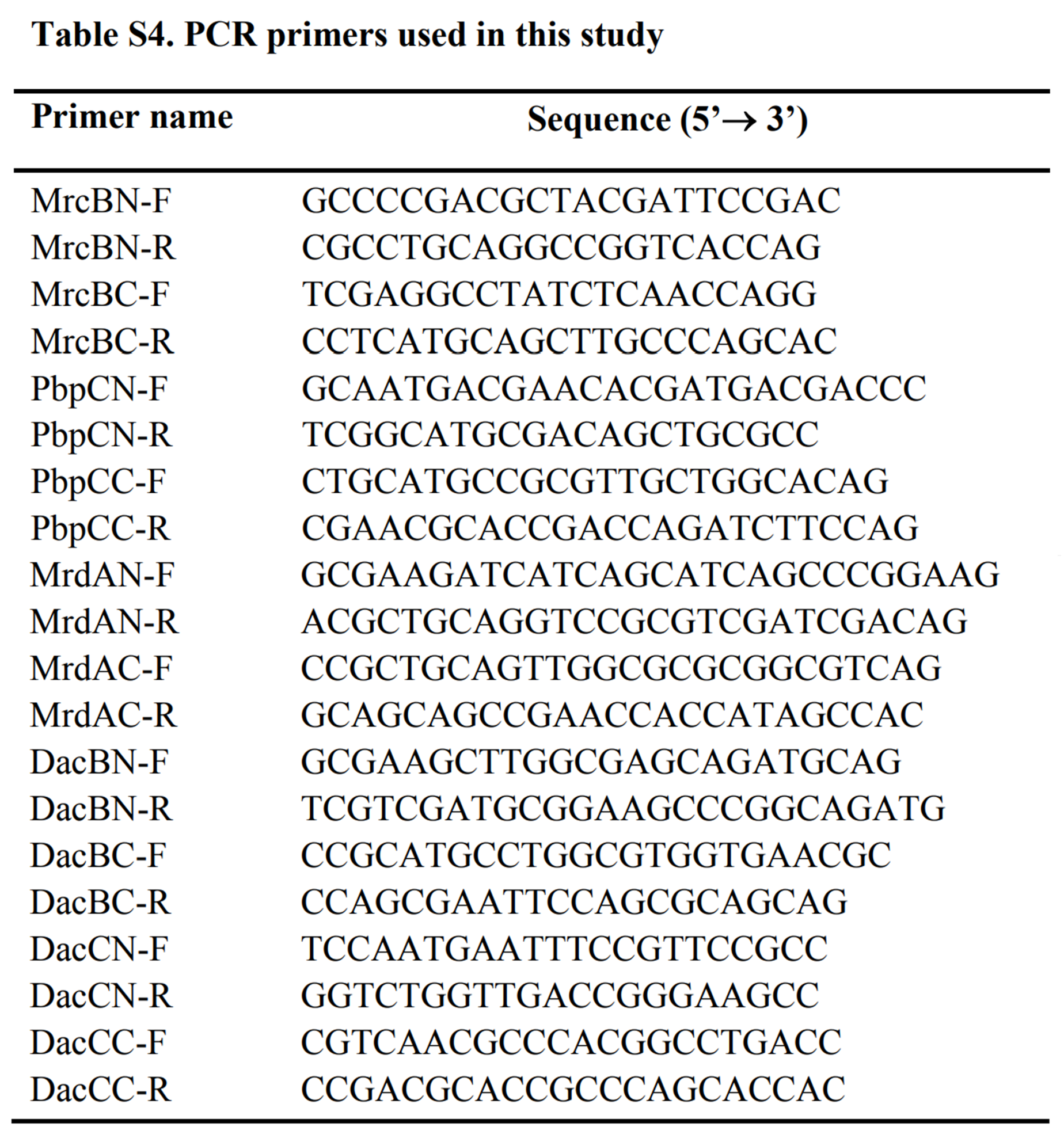

Supplement: TABLE S4 [file sys004172128st4.tif]
